# Diverging in Vivo Immune Response Patterns After Oral Food Challenge in Peanut Allergic Adults

**DOI:** 10.1111/cea.70264

**Published:** 2026-02-25

**Authors:** Florent Fauchère, Aikaterina Alexiou, Veronika Höfer, Kirsten Beyer, Sabine Dölle‐Bierke, Andreas Thiel, Margitta Worm, Julian Braun

**Affiliations:** ^1^ Si‐M / “Der Simulierte Mensch” a Science Framework of Technische Universität Berlin and Charité – Universitätsmedizin Berlin Germany; ^2^ BIH Center of Immunomics, Charité ‐ Universitätsmedizin Berlin, Corporate Member of Freie Universität Berlin and Humboldt‐Universität Zu Berlin Germany; ^3^ Division of Allergy and Immunology, Department of Dermatology, Venereology and Allergy Charité ‐ Universitätsmedizin Berlin, Corporate Member of Freie Universität Berlin and Humboldt‐Universität Zu Berlin Germany; ^4^ Department of Pediatric Respiratory Medicine, Immunology and Critical Care Medicine Charité ‐ Universitätsmedizin Berlin, Corporate Member of Freie Universität Berlin and Humboldt‐Universität Zu Berlin Germany

**Keywords:** adaptive immunity, B cells, food allergy, in vivo immune response, oral food challenge, peanut allergy, Tcells

## Abstract

**Background:**

Peanut (PN) allergy is an IgE‐mediated hypersensitivity with a deviated adaptive immune response dominated by IgE‐producing B cells and type 2 T cells (Th2). The in vivo immune response upon antigen encounter, for example, after oral food challenge (OFC), has not been investigated over prolonged timeframes.

**Methods:**

Here, we investigated the kinetics of allergen‐specific adaptive immune responses in PN‐allergic and non‐allergic adults before OFC (d0) and thereafter at d7, d14, d21, d28 by determining PN‐specific CD40L^+^4‐1BB^+^ conventional (Tconv) and FoxP3^+^ Helios^+^ CD40L^−^4‐1BB^+^ regulatory T cells (Treg), Ara h 2‐specific B cells and PN‐specific sIgE and sIgG4 antibody levels.

**Results:**

In PN‐allergic donors, PN sIgE levels increased until d28 after OFC, but to a variable extent. This variability was associated with a differential increase of Ara h 2^+^ B cells at d7, based on which we identified strong and weak responders (SR and WR). SR exhibited a more pronounced increase of PN sIgE, stronger in vivo activation of T and B cells (HLA‐DR^+^, Ki‐67^+^, CD21^−^), as well as prominent induction of the low‐affinity IgE receptor CD23 in Ara h 2^+^ B cells. In contrast, WR exhibited strong activation of PN‐specific Tregs at d7. At baseline, SR and WR differed regarding Ara h 2^+^ memory B cells and frequencies of Th2 and type 2‐driving Helios‐expressing PN‐specific Tconv. Minor changes in Th1 cells were also observed in non‐allergic donors.

**Conclusions:**

Our results indicate differential adaptive immune responses towards OFC in PN‐allergic adults. In summary, SR displayed a more allergy‐maintaining immune response, while WR displayed a rather suppressive immune response. Underlying responsible factors such as genetics, as well as clinical consequences, will require further investigation in larger cohorts.

## Key Messages

- Peanut‐allergic adults display differentially strong immune responses to oral food challenge.
- Strong responders (~1/3 of donors) display pronounced B and T cell activation and peanut‐specific IgE increase.
- Weak responders are characterised primarily by early peanut‐specific Treg activation.

## Graphical abstract

After the oral food challenge, peanut (PN)‐allergic adults were monitored over 4 weeks, which revealed diverging immune response patterns, with strong responders (SR) exhibiting heightened Ara h 2^+^ B cell expansion, PN sIgE induction and increasing PN‐specific Tconv. Weak responders (WR) had more activated PN‐specific Tregs. This variability in the immune response may have clinical implications.

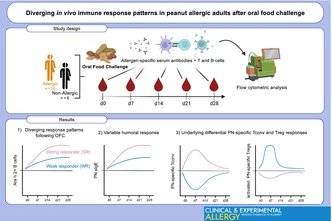

## 1 Introduction

Food allergy is an expanding global medical problem [1]. Peanut (PN) is a common elicitor of food allergy [2, 3]. The major allergens in PN are Ara h 1–18, with Ara h 2 being the immunodominant allergen [4]. Allergic sensitization involves breaking of immune tolerance, leading to allergen‐specific B cells producing IgE, T helper (Th)2 cells secreting IL‐4, IL‐5 and/or IL‐13 and follicular Th cells [5, 6, 7, 8, 9]. Regulatory T cells (Tregs) can become dysfunctional in allergic conditions [10]. Abundance and phenotypes of allergen‐specific T and B cells are critical in the pathophysiology of food allergies, but the in vivo response to allergen ingestion has not yet been investigated in detail.

The double‐blind, placebo‐controlled oral food challenge (OFC) is the gold standard for food allergy diagnosis [11], but also represents a model of allergen ingestion to study the in vivo immune response. Few studies have investigated the allergen‐specific lymphocyte behaviour 1–4 h post‐OFC in vivo using mass cytometry or RNA‐sequencing and observed activation of allergy‐associated immune cells and signalling cascades [12, 13, 14]. However, responses of the adaptive immune system evolve over longer timeframes, e.g., the re‐activation of T and B lymphocytes is detectable in peripheral blood around 7 days after antigen exposure, predominantly as increasing frequencies or upregulation of activation markers, such as HLA‐DR, CD38 or Ki‐67 [15, 16]. Thus, extended monitoring after allergen exposure may provide novel insights into allergic immune responses.

**FIGURE 1:**
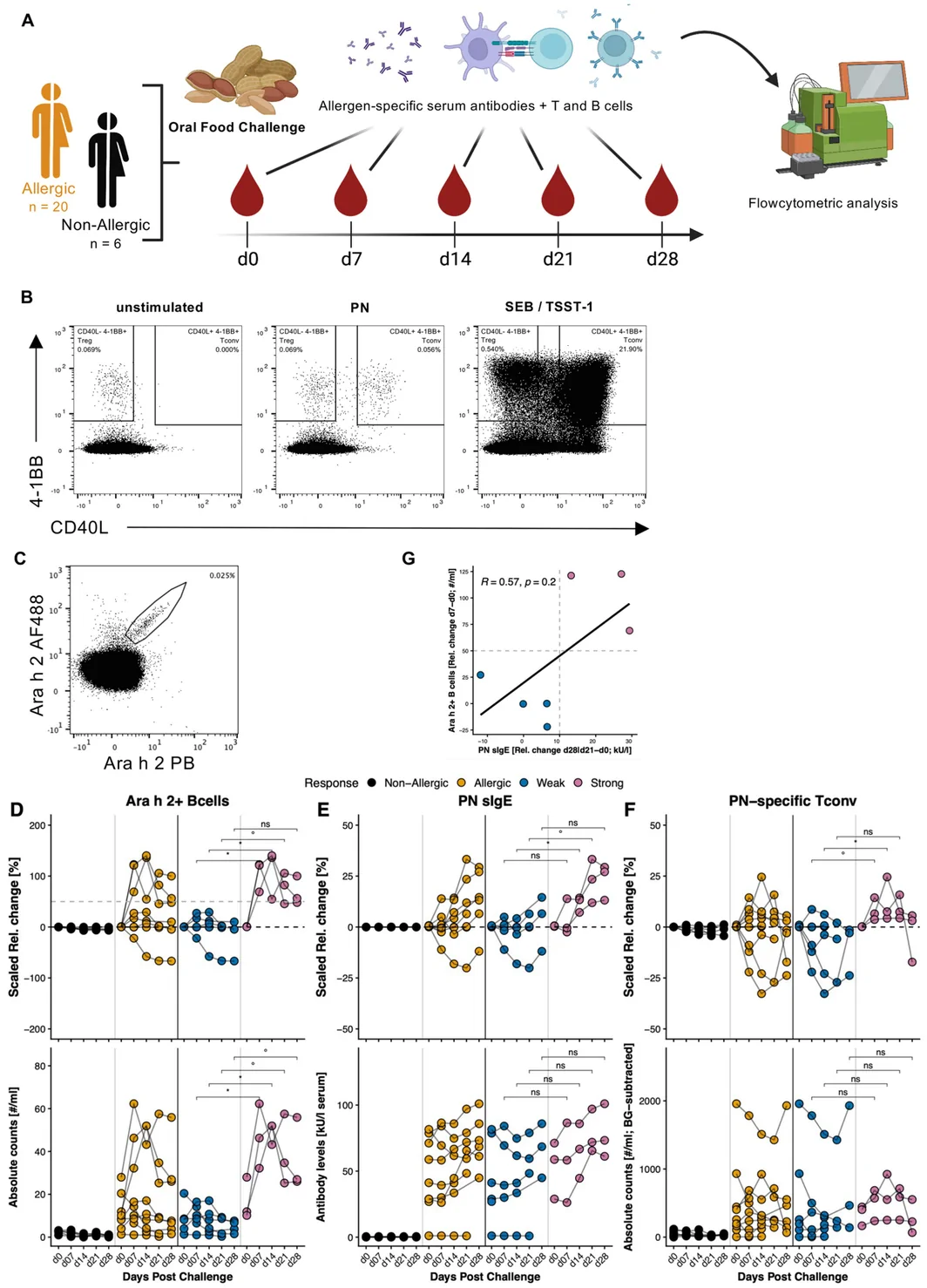
Identification of diverging response patterns. (A) Study design. PN allergic (*n* = 20) and non‐allergic donors (*n* = 6) underwent OFC, blood was sampled before (d0) and weekly after OFC (d7, d14, d21, d28) and antibody levels as well as T and B cell phenotypes were assessed (see Table 1 for details on sampling time points per donor). (B) PBMCs were stimulated with PN extract to assess PN‐specific T cells by staining of activation markers CD40L and 4‐1BB (see also Figures S2 and S3). (C) B cells were dual labelled with fluorescently coupled Ara h 2 (see Figure S3). (D–F) Major adaptive immune parameters depicted as Scaled Rel. change (upper row) and absolute counts of Ara h 2+ B cells and PN‐specific Tconv or antibody concentration in kU/l. (D) Allergic donors (yellow, *n* = 9) were separated according to Ara h 2+ B cells increase at d7 being > 50% (grey dashed line) into early response groups, Weak, WR (blue, *n* = 6) and Strong, SR (pink, *n* = 3), respectively. Statistical comparisons performed using Wilcoxon test (°*p* < 0.1; *p < 0.05; **p < 0.01; ***p < 0.001; ns, not significant). G: Correlation of Ara h 2+ scaled rel. change at d7 with PN sIgE scaled rel. change at d21 or d28 separates WR from SR. Correlation coefficient R and significance level p of Pearson correlations are shown in the graph. Regression line in black.

In this study, we took advantage of OFC as controlled allergen exposure to monitor the in vivo response kinetics of allergen‐specific B and T cells and antibodies in PN‐allergic and non‐allergic adults over the course of 4 weeks. 20 PN‐allergic participants of the TINA study and 6 non‐allergic donors were included [17] (Table 1). Upon enrolment, the allergy status was confirmed by OFC, and blood samples were taken before OFC (d0) and over 4 weeks (d7, d14, d21, d28) (Figure 1A). At all timepoints, we obtained (1) serum to assess total IgE, PN‐ and Ara h 2‐specific sIgE and sIgG4, (2) whole blood for absolute counting of CD4^+^ T cells and B cells and (3) PBMCs to characterise PN‐specific T and Ara h 2‐specific B cells (Figure S1–S4). PN‐specific T cells were determined by stimulation with PN extract followed by flow cytometric identification of antigen‐specific conventional (Tconv, CD40L^+^ 4‐1BB^+^) and regulatory (Treg, CD40L^−^ 4‐1BB^+^) CD4^+^ T cells. Unstimulated controls served to calculate background‐subtractions of frequencies and counts of PN‐specific Tconv (Figure 1B). Ara h 2^+^ B cells were identified by dual‐colour labelling with purified, fluorescently‐labelled Ara h 2 (Figure 1C). Dynamic changes are displayed as relative change at all sampling timepoints (d7–d28) as percentage of the baseline value (d0), adjusted for the donor's magnitude by multiplication with a baseline normalisation factor (scaled rel. change, see Figure S5).

**TABLE 1:** Demographics and clinical parameters for separated cohorts. SPT, Skin Prick Test; OFC MTD dose, Maximum Tolerated Dose (given in dose steps); OFC MTD mg, Maximum Tolerated Dose (given in mg protein); OFC Sampson, Sampson score for reaction severity.

Donor ID	Age [years]	Sex	Disease duration [years]	PN sIgE [kU/L]	SPT	OFC MTD dose	OFC MTD mg	OFC Sampson	Sampling timepoint				
				Mean (range)									
	Mean (range)	(F:M)	Mean (range)		[mm wheal size]				d0	d7	d14	d21	d28
Allergic donors – weak responders (WR)													
Weak responders (WR)	29.8 (19–40)	6:13	25.9 (7–40)	30.1 (0.21–98.5)	10	3.2	78.5	3.8					
A1	34	M	31	1.27	14	2	12	3	x	x	x	x	
A2	30	M	24	34.3	7	3	43	2	x	x	x		x
A3	31	M	26	29.8	7	4	137	4	x	x	x		x
A4	28	M	27	59.9	9.5	4	137	4	x	x	x	x	
A6	31	F	unknown	98.5	21	3	43	4	x	x	x	x	x
A8	33	M	29	81.6	13	1	3	4	x	x	x	x	x
A10	32	M	26	43.8	8	4	137	4	x	x			
A11	19	F	unknown	3.33	5.5	3	43	3	x	x			
A12	40	F	40	2.13	16	4	137	4	x	x			
A13	28	F	7	1.35	11	2	12	4	x	x			
A17	31	F	29	5.99	9	4	137	4	x	x			
A18	24	M	22	29	9	3	43	4	x	x			
A19	26	F	24	0.21	2	4	137	5	x	x			
Allergic donors – strong responders (SR)													
Strong responders (SR)	30.3 (22–39)	2:5	25.9 (20–33)	49.1	13	3.6	339	3.9					
A5	22	M	19	51.7	8.5	3	43	4	x	x	x	x	x
A7	28	F	26	110	7.5	1	3	4	x	x	x	x	x
A9	32	M	20	58.8	16	3	43	4	x	x	x	x	x
A14	39	F	31	1.47	16	6	1378	4	x	x			
A15	31	M	27	110	15	2	12	4	x	x			
A16	26	M	25	8.3	10	5	447	4	x	x			
A20	34	M	33	3.4	21	5	447	3	x	x			
Non‐allergic donors													
Non‐allergic Donors	32.5 (30–35)	1:5	—	0.1	—	8	4500	—					
H1	35	F	—	0.02	—	8	4500	—	x	x	x	x	x
H2	33	M	—	0.03	—	8	4500	—	x	x	x	x	
H3	30	F	—	0.02	—	8	4500	—	x	x	x	x	x
H4	30	F	—	0.02	—	8	4500	—	x	x	x	x	x
H5	32	F	—	0.02	—	8	4500	—	x	x	x	x	x
H6	35	F	—	0.5	—	8	4500	—	x	x	x	x	x

## 2 Materials and Methods

### 2.1 Participants and Ethics

Eligible participants gave informed consent to participation in the randomised clinical trial TINA (tolerance induction through non‐avoidance to prevent persistent food allergy); Trial‐ID: DRKS00016764, currently conducted at the Charité Universitätsmedizin Berlin, Germany [17]. The study was approved by the local ethics committee (Charité Universitätsmedizin, EA2/033/19 and EA2/304/21). Participants were recruited by the Department of Dermatology, Venereology and Allergy, Division of Allergy and Immunology, in accordance with the principles of the Declaration of Helsinki, were ≥ 18 years, with a known primary peanut allergy (allergic) or no food allergy (non‐allergic). Subject characteristics are summarised in Table 1.

### 2.2 Oral Food Challenge and Blood Sampling

An oral food challenge was performed as described elsewhere [17]. In brief, roasted, defatted peanut flour was administered at seven semi‐logarithmic titration steps cumulating to 3 mg, 12 mg, 43 mg, 137 mg, 447 mg, 1378 mg and 4481 mg of food protein at 30‐min intervals until objective, allergic symptoms were observed. If no symptoms occurred, the patient received a cumulative dose of ≙ 4.5 g (step 8). Severity of objective immediate‐type allergic reactions was ranked using the modified Sampson score [18]. Non‐allergic donors ingested the cumulative dose at once, instead of incremental doses.

Blood was taken before OFC (d0) and at d7, d14, d21 and d28 after OFC (Figure 1A). Venous blood was collected in Vacuette lithium heparin tubes (Greiner Bio‐one), and PBMCs were isolated by gradient density centrifugation (Leucosep tubes, Greiner; Biocoll, Bio&SELL) and directly used for flow cytometric analyses.

### 2.3 Lymphocyte Count in Whole Blood

Whole blood (50 μL) was stained with fluorochrome‐conjugated antibodies (Table S1) for 20 min with 1 mg/mL Beriglobin (CSL Behring), a human antibody mix reducing unspecific staining. Erythrocytes were lysed for 30 min at 4°C (Erythrocyte lysis buffer, Qiagen) and stopped by the addition of EDTA. All samples were measured without centrifugation on a MACSQuant Analyser 16 (Miltenyi), which enables absolute counting (# cells/ml). Instrument performance was monitored before measurement with Rainbow Calibration Particles (BD). Flow cytometry data were analysed (Figure S1) using FlowJo 10 (BD).

### 2.4 Allergen‐Specific B Cells

B cells were isolated from PBMCs using CD19 microbeads (Miltenyi) and LS columns (Miltenyi). Surface staining was performed for 30 min with Ara‐H‐2 (Indoor biotechnologies) conjugated with PacificBlue or AlexaFluor 488 (Invitrogen), fluorochrome‐conjugated antibodies and 1 mg/mL Beriglobin (CSL Behring) (Table S1). During the last 5 min of incubation, Zombie Yellow fixable viability staining (Biolegend) was added. Fixation and permeabilization were performed with eBioscience FoxP3 fixation and PermBuffer (Invitrogen) according to the manufacturer's protocol, and intracellular staining was carried out for 30 min. Measurement was performed as described above (gating strategy in Figure S2).

### 2.5 Allergen‐Specific T Cells

Freshly isolated PBMCs depleted from CD19^+^ cells were cultivated at 1 × 10^7^ PBMCs/ml in RPMI 1640 medium (Gibco) supplemented with 10% heat‐inactivated AB serum (Pan Biotech), 100 U/mL penicillin (Biochrom), 0.1 mg/mL streptomycin (Biochrom). Stimulations were conducted with 1 μg/mL purified anti‐CD28 (clone CD28.2, BD) and either with PN extract (prepared as described previously [19], 50 μg/mL), with PBS (unstimulated control) or 1.5 mg/mL SEB and 1 mg/mL TSST1 (Sigma‐Aldrich) and incubated at 37°C, 5% CO_2_ for 18 h. After 2 h, 10 μg/mL brefeldin A (Sigma‐Aldrich) was added, inhibiting vesicle transport and cytokine secretion and enabling intracellular cytokine staining. Stimulations were stopped by incubation in 2 mM EDTA for 5 min. Surface and intracellular staining (Table S1) and measurement were performed as described for allergen‐specific B cells. Gating strategy illustrated in Figures S3 and S4.

### 2.6 Serum Antibody Measurement

Serum antibodies (sIgE and sIgG4) specific for full peanut extract (f13) or Ara h 2 (f423) were determined using the ImmunoCap Phadia 200 immunoassay according to the manufacturer's instructions (Thermo Fisher Scientific). The cut‐off level for sIgE was set at 0.35 kU/l. Detection of sIgE, sIgG4, and total IgE included additional quality controls (low‐, medium‐, and high‐concentrated).

### 2.7 Data Processing and Statistical Analysis

Clinical data was collected and managed using REDCap electronic data capture tools hosted at Charité. Clinical data and laboratory results were compiled and processed and statistically analysed using R (version 4.0.3) and RStudio (1.3.1093). To capture dynamic regulations in longitudinally assessed parameters, three derivatives were calculated (Figure S5): (1) “Delta to d0”, the difference of any timepoint to d0; (2) “Rel. change”, the change relative to d0, i.e., the delta divided by the baseline value; (3) “Scaled Rel. change”, the Rel. change was normalised per parameter by multiplication with a normalisation factor, generated across baseline values. A 50% increase (scaled rel. change) of Ara h 2+ B cells at d7 was utilised as a threshold to separate allergic donors into strong responders (SR, > 50%) and weak responders (WR, < 50%). Non‐parametric statistical comparisons were performed (Wilcoxon and Spearman correlation), and results are displayed as *p*‐values for correlation analyses and as asterisks for Wilcoxon tests (°*p* ≤ 0.1; *p ≤ 0.05; **p ≤ 0.01; ***p ≤ 0.001).

## 3 Results

### 3.1 Identification of Diverging Early Response Types

First, we analysed PN or Ara h 2 sIgE, Ara h 2‐specific B cells and PN‐specific Tconv in allergic and non‐allergic donors with complete kinetics (*n* = 9, d0–d28, Table 1). Over the 4 weeks after OFC, PN sIgE levels increased in four of nine allergic donors by d21 and in five of nine allergic donors by d28 (Figure 1E). An increase in Ara h 2^+^ B cells at d7 was associated with and thus preceded this sIgE increase at d28 (Figure 1D,G). Therefore, we separated the allergic cohort based on the scaled rel. change of Ara h 2^+^ B cells at d7 into two response types, weak responders (< 50%, blue, WR, *n* = 6) and strong responders (> 50%, pink, SR, *n* = 3) (Figure 1D). In SR, Ara h 2^+^ B cell counts rapidly increased by d7 and d14 and levelled at 50%–100% above baseline by d28, suggesting an expanded pool of Ara h 2‐specific B cells 1 month after OFC. In contrast, WR exhibited only a minor increase below 50% above baseline at d7 and d14, and values returned close to baseline by d28. These different B cell dynamics translated into differently pronounced increases in PN sIgE levels (Figure 1E). In SR, PN sIgE increased 12%–33% above baseline levels until d28, while it decreased or only marginally increased (ranging from −20% to 14%) in WR responders. Furthermore, PN‐specific Tconv at d7 were increased in SR (ranging from 3.9% to 13.6%), significantly different from the decrease observed in WR (ranging from −20.8% to 4.7%) (Figure 1F).

Altogether, we identified weak and strong allergen‐specific immune responses in allergic donors, based on transitional heterogeneous changes of Ara h 2^+^ B cells, PN sIgE and PN‐specific Tconv in allergic donors, observed as early as 1 week after allergen ingestion (OFC).

### 3.2 Early Response Types Are Reflected in B Cell Activation and Differentiation

**FIGURE 2:**
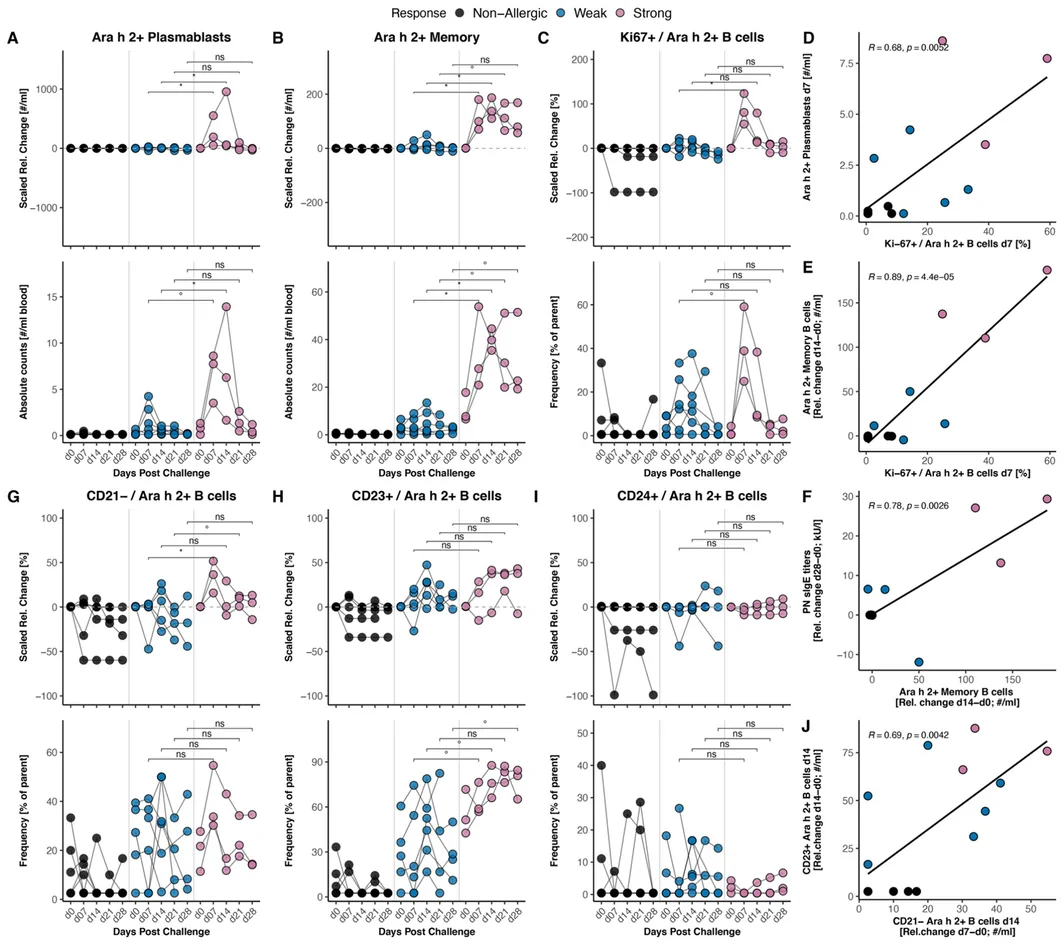
Response types are manifested in B cell subsets and activation. (A–C), (G–I) Scaled rel. change (upper plot) and absolute counts or frequency of parent (lower plot) of indicated cell population over time displayed for Non‐Allergic (black, *n* = 6), allergic Weak (blue, *n* = 6) and Strong (pink, *n* = 3) responders. Statistical comparisons performed using Wilcoxon test (°*p* < 0.1; *p < 0.05; **p < 0.01; ***p < 0.001; ns, not significant). (D–F, J) Correlations of indicated parameters. Correlation coefficient R and significance level p of Pearson correlations are shown in graph. Regression line in black.

The stronger induction of PN sIgE in SR should be mirrored by B cell activation, expansion and differentiation. Indeed, SR exhibited significantly higher frequencies of proliferating, in vivo activated Ki67^+^ among Ara h 2^+^ B cells at d7 and d14 (Figure 2A), followed by significant expansion of Ara h 2^+^ plasmablasts at d7 and d14 (Figure 2B) and Ara h 2^+^ Memory B cells until d28 (Figure 2C). Higher frequencies of proliferating (Ki‐67^+^) Ara h 2^+^ B cells at d7 correlated well with Ara h 2^+^ Plasmablasts at d7 (Figure 2D) and with the induction of Ara h 2^+^ Memory B cells until d14 (Figure 2E), which in turn correlated with the increase of PN sIgE concentrations until d28 (Figure 2F). We also observed a significant increase of CD21^−^ Ara h 2^+^ B cells in SR (Figure 2G). CD21, the complement receptor 2, is a co‐receptor of CD19 and the B cell receptor and lowers activation thresholds, thus rendering B cells more responsive [20, 21, 22, 23]. Thus, CD21^−^ B cells are regarded as refractory.

Furthermore, CD23 was induced continuously in SR, but intermittently in WR (Figure 2H). CD23, the low‐affinity IgE receptor, on allergen‐specific B cells, identifies a population of Memory B cells that class‐switch from IgG1 to IgE and thus maintain the allergy‐driving B cell pool and IgE production [24, 25, 26, 27, 28]. The increase of CD23^+^ Ara h 2^+^ B cells at d14 correlated strongly with the rel. change of activated, CD21^−^ Ara h 2^+^ B cells by d7 (Figure 2J), thus underlining this recently described differentiation route. Finally, CD24^+^ B cells did not appear to be regulated, but tended to be more abundant in three WR as compared to SR (Figure 2I). In WR, the elevated frequency of CD24^+^ B cells, described as regulatory B cells (Bregs), may reflect the suppressed activation of B cells [29]. Among bulk B cells, these CD21^+^, CD23^+^ and CD24^+^ phenotypes did not differ, highlighting the antigen‐specific nature of these dynamics (Figure S7).

Altogether, SR exhibited a marked increase in activated and proliferating Ara h 2^+^ B cells, plasmablasts and memory B cells, as compared to WR.

### 3.3 Validation of Diverging Response Patterns and Differences in T Cell Responses

**FIGURE 3:**
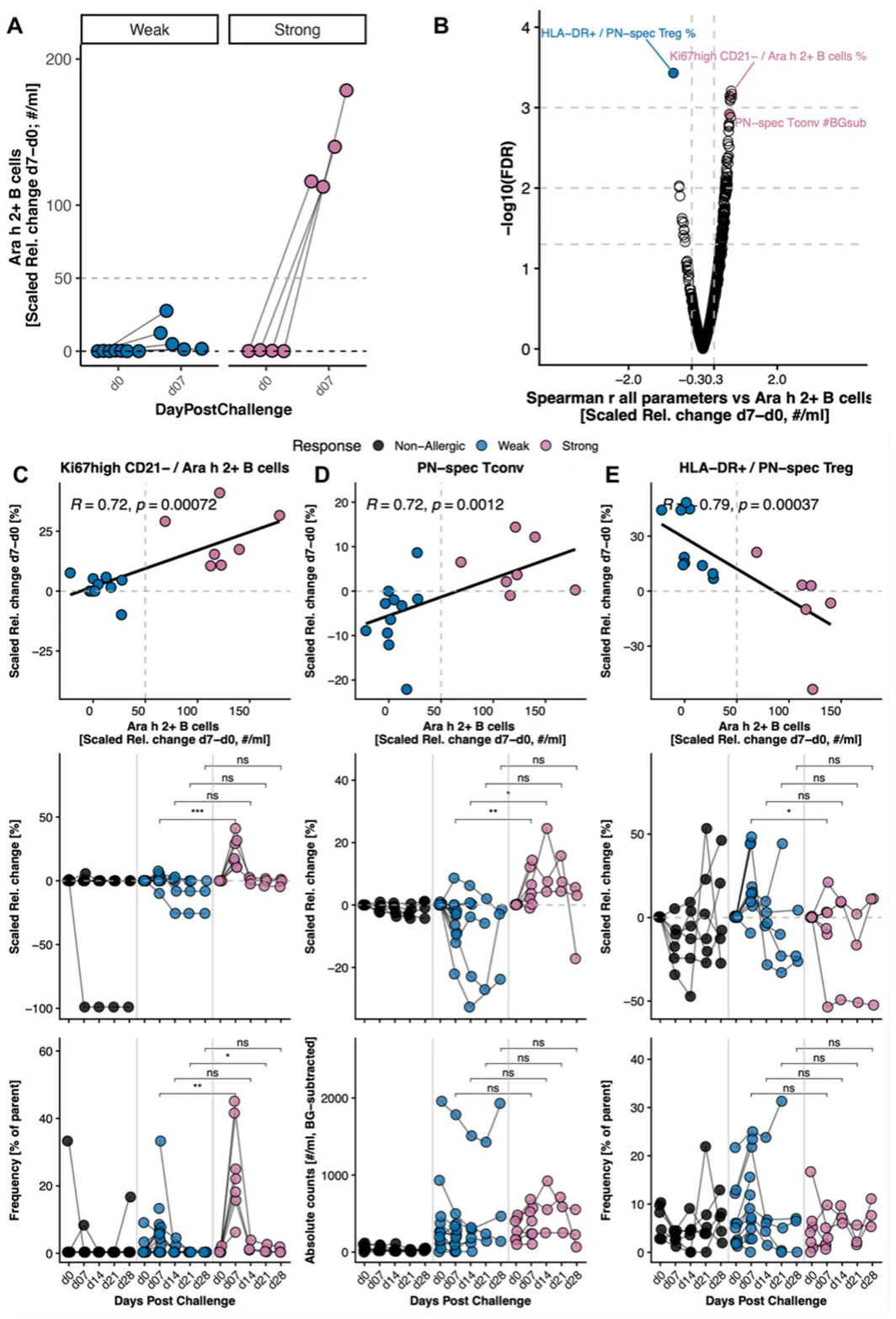
Validation of early response types reveals differential T cell responses. (A) Additional donors (short kinetics) sampled at d0 and d7 (*n* = 11) were separated into weak (*n* = 7) and strong (*n* = 4) according to the scaled rel. Change of Ara h 2+ B cell counts at d7 below or above 50% (indicated by dashed, grey line). (B) Spearman correlation of Ara h 2+ B cell scaled rel. change at d7 with scaled rel. changes of all parameters at d7 in the cohort of allergic donors (*n* = 20). (C–E) Correlation of scaled rel. change with scaled rel. change of Ara h 2+ B cells at d7 (top panel), scaled rel. change (middle panel) and raw data as frequency of parent (lower panel) of the indicated parameters. Statistical comparisons performed using Wilcoxon test (°*p* < 0.1; *p < 0.05; **p < 0.01; ***p < 0.001; ns, not significant). Spearman correlation (correlation coefficient R and significance level p shown in graph). Regression line in black.

Since B cell responses are coordinated by antigen‐specific T cells, we next investigated T cell responses of SR and WR. To extend the cohort, short kinetic donors were included (*n* = 11, Figure 3A and Table 1), yielding a total of 7 SR and 13 WR.

The most co‐regulated parameter (Figure 3B) was Ki67^high^CD21^−^/Ara h 2^+^ B cells, being strongly increased in SR (Figure 3C), confirming their stronger B cell response. Furthermore, PN‐specific Tconv were significantly increased at d7 in SR, but stayed constant or decreased in WR (Figure 3D). Both observations are in line with findings from the smaller cohort (Figures 1E, 2D,F), thus underlining the diverging response patterns. The only parameter significantly upregulated in WR was HLA‐DR^+^ among PN‐specific Tregs, with a sharp and transient increase at d7, indicating in vivo activation, in comparison to a decrease in SR (Figure 3B,E and Figure S8F–K). WR also exhibited a significantly higher induction of CCR4 among PN‐specific Tregs but not other T cell subsets (Figure S8L–Q). This indicates a pronounced activation and recruitment of PN‐specific Treg in WR early in the immune response, with suppressive effects that may contribute to the weaker response.

At helper T cell (Th) subset level, frequencies of Th1 stayed rather constant, and no in vivo activation was observed in allergic donors (Figure S8A,H). In contrast, Th2 were heterogeneously regulated in allergic donors, and activation of Th2 appeared more pronounced in SR at d7 as compared to WR (Figure S8B,I). Strikingly, only WR exhibited a sharp increase in IL‐5^+^ Th2 at d7 (Figure S8C). We also observed minor changes among non‐allergic donors, with Th1 being increased in two non‐allergic donors, while almost no change appeared for Th2 (Figure S8A,B). Heterogeneous dynamics were also observed among Th17 cells and Th0 (Figure S8D,E,J).

In summary, the diverging response patterns can also be dissected at the T cell level at d7 after OFC, based on increases or decreases and activation of PN‐specific T helper subsets or Tregs.

### 3.4 Baseline Characteristics of Response Types

**FIGURE 4:**
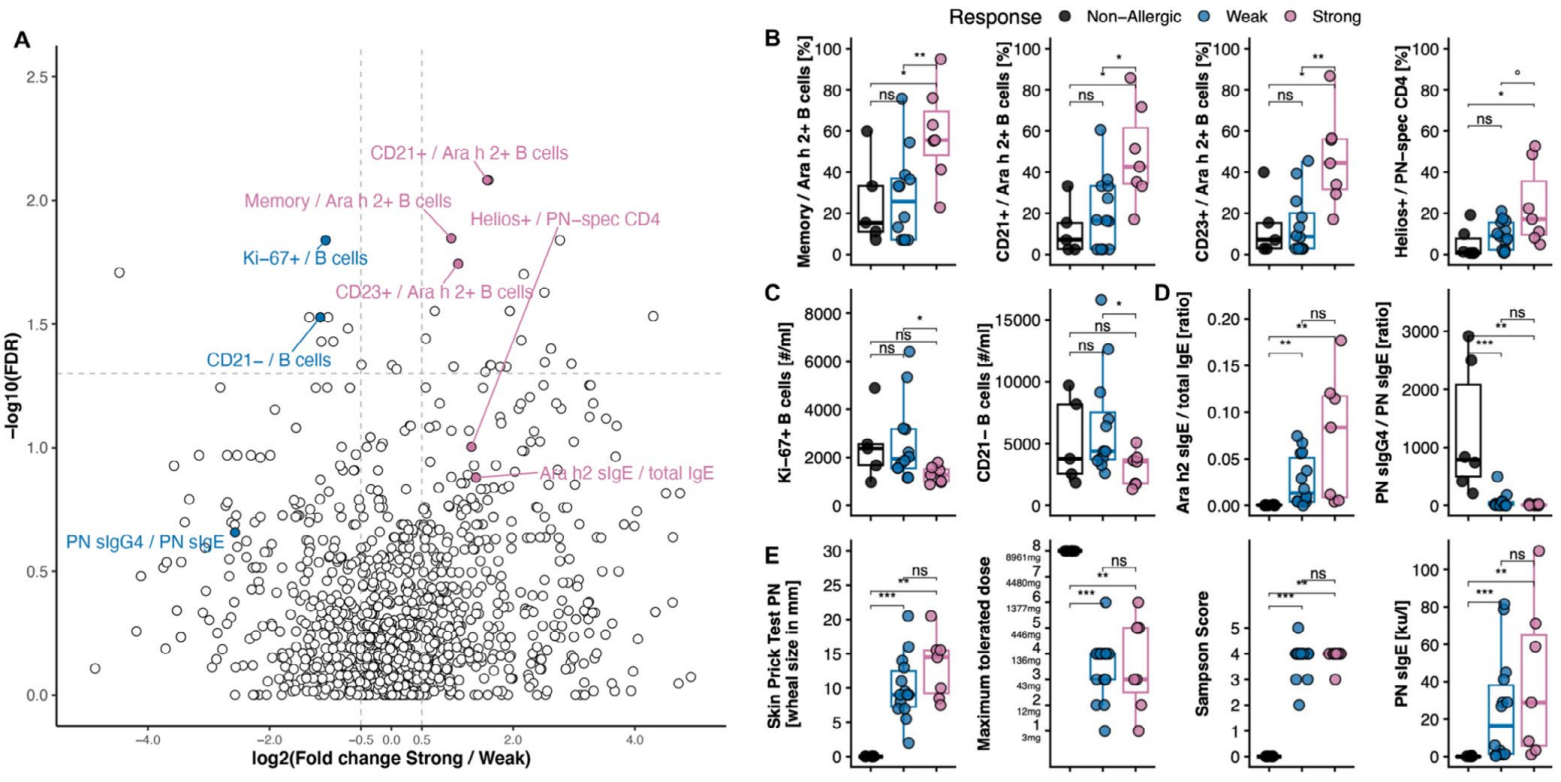
Baseline differences between early response types. (A) Baseline parameters were compared between response groups of WR (*n* = 13) and SR (*n* = 7). The false discovery rate (FDR, −log10 (p) from Wilcoxon test) and the log2 Fold change of the means between SR and WR are displayed. Not‐highlighted, significant parameters represent parameters directly associated with the highlighted ones, such as counts or frequencies of the same cell population. B‐E: Box plots showing baseline differences being elevated in SR (B), in WR (C) and serological differences (D). Relevant clinical parameters are additionally displayed (E). Statistical comparisons performed using Wilcoxon test (°*p* < 0.1; *p < 0.05; **p < 0.01; ***p < 0.001; ns, not significant).

We next investigated baseline characteristics of SR and WR by comparing the fold change between WR and SR and the statistical significance of each parameter (Figure 4A). Most significantly, SR exhibited more memory among Ara h 2^+^ B cells, which also harboured more CD21^+^ and CD23^+^ cells (Figure 4B). Further, Helios, a transcription factor associated with Th2 cells [30, 31], was more abundantly expressed among PN‐specific Tconv in SR. In contrast, WR tended to have more Ki‐67^+^ and CD21^−^ among bulk B cells independent of antigen specificity (Figure 4C), indicating activated B cells, potentially originating from ongoing immune responses against past or current infections. These B cell subsets positively correlated with HLA‐DR^+^ CD4^+^ T cells at baseline, another marker for ongoing immune responses, further corroborating this notion (Figure S9). In addition, SR had higher ratios of Ara h 2 sIgE among total IgE, while WR had slightly higher ratios of PN sIgG4 relative to PN sIgE (Figure 4D). Although not significant, this may provide additional reasoning for the observed diverging response patterns. No differences in clinical parameters were observed, neither for skin prick Test, the OFC maximum tolerated dose, the modified Sampson severity score [32], nor PN sIgE levels (Figure 4E). Additional clinical parameters also revealed no differences between WR and SR (Data S1).

**FIGURE 5:**
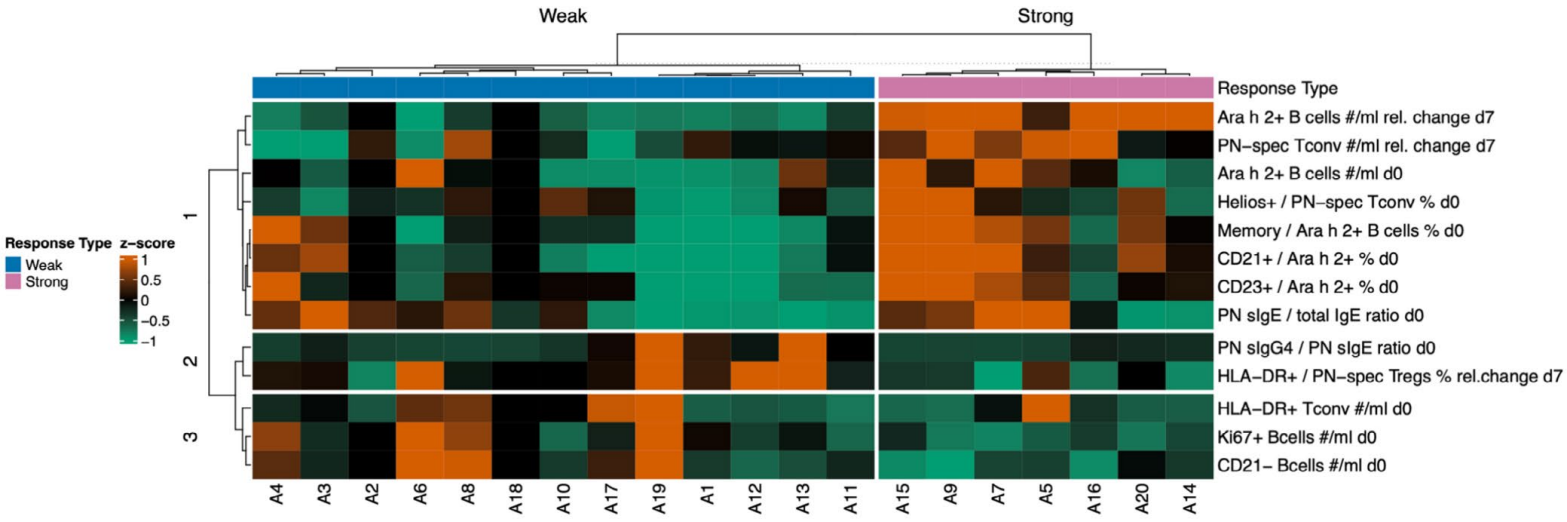
Summary of major differences between early response types. Heatmap displays major parameters involved in diverging response types clustering together in (1) allergy‐promoting parameters, (2) allerge‐nspecific immune suppression, and (3) immune state‐associated parameters.

Taken together, WR and SR differed at baseline in subsets of antigen‐promoting B and T cells, but also general immune activation states and immunosuppressive parameters (summarised in Figure 5).

## 4 Discussion

Herein, we describe the in vivo allergen‐specific adaptive immune responses triggered by OFC in PN‐allergic and non‐allergic adults over 4 weeks. PN‐specific cellular and humoral immune responses appeared consistently regulated across multiple assay systems, which lends credibility to the described dynamics.

Most strikingly, PN and Ara h 2 sIgE increased in most allergic donors until d28 to different extents, ranging from 5% to 33% above baseline. We explain this variability with two underlying diverging early response patterns, namely “Weak” (WR) and “Strong” (SR), characterised by less or more than 50% increase of Ara h 2^+^ B cell counts at d7. SR further exhibited more pronounced activation and proliferation of Ara h 2^+^ B cells, i.e., intermittent increase of Ki‐67^+^ and CD21^−^ cells, followed by a clear expansion of plasmablasts and memory B cells at d7 and d14, and a sustained increase of CD23^+^ among Ara h 2^+^ memory B cells. Further, SR exhibited an increase in PN‐specific Tconv by d7, which was associated with PN‐specific Th2 activation and expansion. In contrast, WR exhibited decreased PN‐specific Tconv at d7, but more activated Tregs. These features characterise the two early response types.

Recent studies with longitudinal comparisons provide some evidence for diverging immune responses after OFC. An increase in PN sIgE following OFC has been reported for oral immune therapies [33]. Increased or decreased PN‐specific T cell frequencies at day 15 after OFC were observed in the placebo group of a clinical trial [34]. Similarly, Wasp venom‐reactive T cells were increased 1 week after allergen exposure in most donors [35]. These few reports support our observations of elevated sIgE and diverging T cell responses following allergen exposure.

The observed baseline differences between WR and SR offer indications for three alternative explanations for the divergent responses (summarised in Figure 5). Firstly, SR displayed more allergen‐specific Memory B cells with allergy‐maintaining phenotypes (CD23^+^, CD21^+^) [22]. In addition, Helios, a transcription factor previously associated with Th2 cells [30], was increased in PN‐specific Tconv of SR, which is in line with the expansion of PN‐specific T cells and higher Th2 frequencies in SR. Accordingly, SR may be explained simply by more abundant allergen‐specific B and T cells at baseline. Secondly, three WR had more in vivo activated B cell subpopulations among bulk B cells (Ki67^+^, CD21^−^) and consistently more activated HLA‐DR^+^ CD4^+^ T cells, which can be interpreted as signs of ongoing immune responses, e.g., following recently contracted infections. This may dampen immune responses against immediately following challenges, i.e., the OFC [36, 37, 38], and thus offers an alternative explanation for the different response types. Thirdly, WR tendentially exhibited higher ratios of PN sIgG4 relative to PN sIgE, which is a marker of tolerance development. IgG4 binds PN antigens, which leads to reduced mast cell activation and immune cell recruitment and thus a weaker response. All three explanations may hold true, which however has to be elucidated in future studies.

We also observed a marginal immune response in non‐allergic donors. According to a recent study, around 50% of non‐allergic donors had antibodies against diverse food proteins [39], which is in line with our data of 3 from 7 non‐allergic donors being IgG4^+^ (Figure S6). The subtle changes of Th1, Th17, and Th0, but not Th2, in response to food components in non‐allergic individuals, are noteworthy effects, relevant for basic immunological understanding [40].

Our study also has weaknesses. Firstly, the patient cohort has limitations. The necessary weekly kinetics are hard to include in most study regimes, due to, e.g., commencing desensitisation therapy shortly after OFC as in the TINA trial. The frequent visitations including blood taking represent further logistical hurdles. Therefore, we retrieved only 9 complete kinetics, and this low sample size often impedes statistical comparisons. Secondly, the applied phenotyping of allergen‐reactive T cells focused on cytokine production (IFN‐γ, IL‐4, IL‐5 or IL‐17) is limited. Many PN‐specific Tconv do not secrete the included cytokines, referred to as “Th0”, which may include IL‐13‐producing Th2, Th9 or Th22 among others. Further, our flow cytometric panels did not include markers for direct identification of Th2A cells (CRTH2, CD27, CD45RB, CD49d, CD161) [41]. These populations have a pivotal role in allergy, by supporting B cell activation and class switching [42, 43, 44, 45, 46]. The abundance of Th2A cells differs in allergic donors and may influence allergic immune responses dramatically [47]. Alternative phenotyping strategies must be utilised in future kinetic experiments to cover all relevant cell types. Thirdly, the applied B cell depletion may disturb T cell activation. Due to low available blood volumes, we separated B cells from PBMCs by magnetic enrichment to enable parallel T cell stimulation and Ara h 2^+^ B cell assessment. B cell depletion indeed reduces the frequency of CD40L^+^4‐1BB^+^, however, both in unstimulated and PN‐stimulated samples (see Figure S10). This effect was resolved by background subtraction, and thus the described longitudinal changes are not affected by B cell depletion. Finally, in our study, allergic donors reacted mainly to the 4th or 5th dose and thus were screening failures in the TINA trial or were randomised into the avoidance arm and did not undergo treatment. Therefore, we cannot determine whether the diverging response types are associated with reaction threshold, symptom severity or treatment success. We speculate that the increase of PN sIgE after OFC could modulate the response to desensitisation therapy when performed in conjunction with treatment initiation. For example, WR with reduced activation of PN‐specific Th2 and more activated HLA‐DR^+^ PN‐specific Tregs may respond better to desensitisation. HLA‐DR^+^ Tregs were demonstrated to be associated with successful desensitisation therapy [48]. Therefore, whether WR may predict desensitisation therapy success has to be assessed in future studies.

## 5 Conclusion

The herein described findings shed light on diverging adaptive immune response patterns in PN allergy upon OFC. These diverging adaptive immune responses may be associated with the aggravation of allergic symptoms or promote the development of tolerance. The described dynamics aid in understanding mechanisms of immune responses in food allergy, which have to be investigated in larger studies with defined patient cohorts.

## Author Contributions

Florent Fauchère, Andreas Thiel and Julian Braun designed the experimental set‐ups. Florent Fauchère performed experiments. Julian Braun, Florent Fauchère and Margitta Worm analysed and interpreted the data. Florent Fauchère, Julian Braun and Margitta Worm wrote the manuscript. Margitta Worm and Kirsten Beyer conceptualised the FOOD@ consortium. Margitta Worm and Kirsten Beyer designed the clinical studies and developed the consortium. Margitta Worm, Kirsten Beyer, Sabine Dölle‐Bierke supervised clinical management and clinical data. Aikaterina Alexiou, Veronika Höfer conducted the OFC and Veronika Höfer, Aikaterina Alexiou, Sabine Dölle‐Bierke are responsible for enrolling participants, clinical data assessment, and bio‐sample acquisition, storage and distribution. All authors read and approved the manuscript.

## Ethics Statement

This study has been reviewed and approved by the ethics committee at the Charité Universitätsmedizin in Berlin and determined that the research complies with all applicable ethical standards and regulations to ensure the protection of human subjects. All procedures are conducted in accordance with the approved protocol, and informed consent was obtained from all participants prior to their involvement.

## Conflicts of Interest

The authors declare no conflicts of interest.

## Supporting information


**Table S1:** Antibody panels for flow cytometry.
**Figure S1:** Gating scheme for the whole blood count panel.
**Figure S2:** Gating scheme for Ara h2‐specific B cells and memory subsets.
**Figure S3:** Gating scheme for antigen‐specific T cells, helper subsets and homing markers.
**Figure S4:** Gating scheme for T cell activation, in vivo activation markers and checkpoint.
**Figure S5:** Derivatives to capture dynamic regulations.
**Figure S6:** Overview of IgG4 serology.|
**Figure S7:** Dynamics of selected bulk B cell populations.
**Figure S8:** Dynamics of relevant T cell subsets.
**Figure S9:** Correlation of activated B cells and T cells points at general immune.
**Figure S10:** Impact of B cell removal on the T cell stimulation assay.

**Data S1:** cea70264‐sup‐0002‐Supinfo1.pdf.

## Data Availability

The data that support the findings of this study are available on request from the corresponding author. The data are not publicly available due to privacy or ethical restrictions.
