# Supplementary material for: Diverging in Vivo Immune Response Patterns After Oral Food Challenge in Peanut Allergic Adults
Source: Clin Exp Allergy. 2026 Feb 25;56(9):1063–75. doi: 10.1111/cea.70264 (PMC13532977; doi:10.1111/cea.70264)
Supplement: Supplementary file 1 [file CEA-56-1063-s001.pdf]

								PN Allergy				PN OFC													
Study ID	OFC Response	Ethnicity	Mode of delivery	Age in years	2. Cardiovascular Diseases	6. Other chronic condition	Have you ever had a parasite infection?	Date of diagnosis	Index reaction	Severity score (modified Sampson Score)	Muraro score	Allergen	Threshold dose (dose step leading to discontinuation of OFC)	Maximum cumulative threshold dose of allergen on final OFC day:	Severity according to modified Sampson Grading	Muraro score	Rescue medication administered?	No Antihistamines	Oral Antihistamines	Antihistamines i.v.	No Glucocorticoides	Glucocorticoides i.v.	ß2 Mimetika	No Epinephrin	Epinephrin i.m.
A1	Weak	Caucasiar	Vaginal	34				01.01.90				Peanut	3	0,04	III	II - mode	Yes			X		X	None	X	
A10	Weak	Caucasiar	Vaginal	32				01.01.95	Jan 08	IV	III - severe	Peanut	5	0,447	IV	II - mode	Yes			X		X	None		X
A11	Weak	Caucasiar	Vaginal	19								Peanut	6	1.378	II	I - mild	Yes		X	X		X	None	X	
A12	Weak	Caucasiar	Vaginal	40				01.01.82				Peanut	5	0,447	IV	II - mode	Yes			X		X	Inhalation		X
A13	Weak	Caucasiar	Vaginal	28		Tonsillectomy		01.06.15				Peanut	3	0,043	IV	I - mild	Yes			X		X	None		X
A17	Weak	Caucasiar	Caesarea	31		Migraine		01.01.94				Peanut	5	0,447	IV	II - mode	Yes			X		X	None		X
A18	Weak	Caucasiar	Vaginal	24				01.01.02	Jan 02	II	I - mild	Peanut	4	0,137	IV	II - mode	Yes			X		X	Inhalation		X
A19	Weak	arabic/ca	Vaginal	26				01.01.00	Jan 00	III	II - moderate	Peanut	5	0,447	V	III - seve	Yes			X		X	Inhalation		X
A2	Weak	Caucasiar	Vaginal	30		Appendectomy		01.01.97	Jan 97	IV	II - moderate	Peanut	4	0,126	II	I - mild	Yes	X			X		None	X	
A3	Weak	Caucasiar	Vaginal	31		appendectomy		01.01.95				Peanut	5	0,447	IV	II - mode	Yes			X		X	Inhalation		X
A4	Weak	Caucasiar	Vaginal	28				01.01.95	Jan 98	IV	II - moderate	Peanut	5	0,447	IV	II - mode	Yes			X		X	None		X
A6	Weak	Caucasiar	Vaginal	31								Peanut	4	0,137	IV	II - mode	Yes			X		X	None		X
A8	Weak	Caucasiar	Vaginal	33				01.01.95	Jan 95	III	II - moderate	Peanut	2	0,012	IV	II - mode	Yes			X		X	Inhalation		X
A14	Strong	Caucasiar	Vaginal	39				01.01.91	Jan 91	I	I - mild	Peanut	7	4.481	IV	II - mode	Yes			X		X	None		X
A15	Strong	Caucasiar	Vaginal	31		varicose veins (righ		01.01.96	Jan 96			Peanut	3	0,043	IV	I - mild	Yes			X		X	None	X	
A16	Strong	Caucasiar	Vaginal	26	Mitral valve prolaps			01.01.98	Jan 98	II	I - mild	Peanut	6	1.378	IV	II - mode	Yes			X		X	Inhalation		X
A20	Strong	Caucasiar	Vaginal	34			Yes	01.06.91				Peanut	6	1.378	III	II - mode	Yes			X		X	None		X
A5	Strong	Caucasiar	Caesarea	22				01.01.03		IV	II - moderate	Peanut	4	0,137	IV	II - mode	Yes			X		X	None		X
A7	Strong	Asian	Vaginal	28				01.01.96	Jan 96	II	I - mild	Peanut	2	0,012	IV	II - mode	Yes			X		X	Inhalation		X
A9	Strong	Caucasiar	Vaginal	32				01.01.04				Peanut	4	0,137	IV	II - mode	Yes			X		X	None		X

			Food Allergies											?, suspected; X, yes; X!, physician confirmed; +, systemic reaction; -, never eaten; $, symptoms improved, S, SIT ongoing																																		
Epinephrin Inhalation	Fluids	O2	2. Chickpea	3. Lentils	4. Pea	Peanut	6. Soy	processed soy tolerated?	lupi	Fenu	1. Almond	2. Brazil nut	Cashew	Hazelnut	5. Macadamia	6. Pistachio	7. Pecan	Walnut	1. Sesame	2. Pine Nuts	sunflower seed, pumkin seed	2. Hen's egg (raw, baked)	3. Fish (any)	4. Shellfishes (any)	Buckwheat	Apple	Peach	Kiwi	Cherry	Celery	Tomato	Carrot	Spices	Herbs	Other {al_fruit_free}	Orange, Citrus	Mango	Melon	Grapes	Pear	Apricot, Nectarinme	Plum	Pineapple		Has the subject any medication being prescribed? (choice=Antihistamines)	Has the subject any medication being prescribed? (choice=Glucocorticoides)	Has the subject any medication being prescribed? (choice=ß2 Mimetics)
	Yes	Yes				X!+					?+	?	?-	?-	?	?	?	?+		?		X!	X!	X!	X!		X	X																	X	X	
	Yes	None				X!+						?	?		?		?																												X	X	
	Yes	None				?+								?		?+	?	?+								X	X																				
	Yes	Yes				X!+	?				?	?	?		?		?			?																									X	X	
	Yes	None				X!+																				X		X																	X	X	
	Yes	None				X!+								X!+									?			X	X		X		X				X	X							X		X	X	X
	Yes	Yes				X!+																						X							X								X		X	X	X
	Yes	Yes				X!+	?											?										X							X		X	X					X		X	X	X
	Yes	None				X!+						?	?-			?	?	?		?								X																	X	X	
	Yes	Yes		?		X!+																																							X	X	
X	Yes	Yes	?	?		X!+					?	?		?+	?	?	?	?		?																									X	X	
	Yes	Yes	?	?+	?+	X!+	?+	X	?		?+	?	?	?	?	?	?	?	?	?						X	X	X	X			X	X			X									X	X	X
	Yes	None				X!+				?		?	?		?	?	?																												X	X	
	Yes	None				X!+						?	?	?	?	?	?			?						X	X	X	X	X		X													X	X	X
	Yes	None				X!+						?	?		?	?	?																												X	X	
	Yes	Yes				X!+	?										?									X	X		X			X			X			X		X	x	X			X	X	
	Yes	Yes				X!+					X!+		X!+	X!+				X!+	X!	?	X		X!																						X	X	X
	Yes	Yes				X!+					?	?	?		?		?	?		?			?			X		X				X													X	X	X
X	Yes	Yes				X!+						?	?+		?	?	?			?																	X	X	X						X		
	Yes	Yes				X!+																																							X	X	

	Other Allergies					Asthma														Eczema							
Has the subject any medication being prescribed? (choice=Epinephrine auto-injector)	Other allergic diseases	Year of diagnosis	If yes, current symptoms? (< 12 months)	Please define other allergic diseases	Year of diagsis	Asthma history	Asthma active	Asthma elicitor Exercise	Asthma elicitor Infection/ cold	Asthma elicitor Animals	Asthma elicitor House dust mite	Asthma elicitor Grass pollen	Asthma elicitor Tree pollen (i.e. hazel, alder, birch	Asthma elicitor Weed pollen (mugwort, ragweed	Asthma elicitor Laughing	If other elicitor of asthma, please specify	Asthma Treatment SABA (short-acting-beta-agonist; e.g., Salbutamol, Feterol, Terbutalin))	Asthma Treatment LABA (long-acting-beta-agonist; e.g., Salmeterol, Formoterol))	Asthma Treatment LABA/ICS (inhaled corticosteroids + long-acting-beta agonist; e.g.; Budesonid/Formoterol))	Eczema	Excema Active	Eczema Treatment Basic care)	Eczema Treatment Antiinflammatory)	If basic care (choice=Without additives)	Basic care With additives (e.g., Polidocal, Tricolsan, Zinc, Glycerin, Urea))	Antiinflammatory Topical steroids (e.g., Advantan, Hydrogalen))	Antiinflammatory Calcineurin inhibitors (e.g., Protopic, Elidel))
X						1990	1990	X		X	X	X					X			1987	1987						
X																				1992	1992						
																				2001	2001						
X	Amoxicillin Type IV reaction	2011		suspected penicillin intolerance	2011															1982							
X						2000														1993	1993	X	X		X	X	
X						2003														1994	1994	X	X		X	X	
X																											
X	Latex allergy	2021				2023	2023										X		X	1997	1997						
X																											
X	suspected allergy to cough medication	2002				2000																					
X	Adeidectomy	2000																		1993							
X	Chronic rhisinusitis		Yes			1998	1998	X	X	X	X	X	X	X	X	X	X		X	1996	1996	X	X		X	X	
X																											
X						1992	1992	X	X	X							X		X	1983	1983	X	X	X	X	X	
X																				1991							
X	Penicillin	2007				2002														1997							
X						1990	1990	X										X		2015							
X	suspected penicillin					2006	2006													2003	2003	X	X	X		X	
X	Latex allergy	2016				2010																					
X																											
