# Supplementary material for: Diverging in Vivo Immune Response Patterns After Oral Food Challenge in Peanut Allergic Adults
Source: Clin Exp Allergy. 2026 Feb 25;56(9):1063–75. doi: 10.1111/cea.70264 (PMC13532977; doi:10.1111/cea.70264)
Supplement: Supplementary file 2 [file CEA-56-1063-s002.pdf]

Supplementary Table S1: Antibody panels for flow cytometry

Whole blood count panel

	**Surface Staining**	
**CD8 Vioblue**	REA734	Miltenyi
**CD14 Viogreen**	REA599	Miltenyi
**CD4 BV605**	RPA-T4	Biolegend
**CD20 FITC**	2H7	Biolegend
**CD45 Percp**	2D1	Biolegend
**CD19 PE-Cy7**	SJ2C1	Biolegend
**CD3 APC**	OKT3	Biolegend

B cell panel

	**Surface Staining**	
**IgD-BV510**	IA6-2	Biolegend
**CD14-BV570**	M5E2	Biolegend
**CD3-BV570**	UCHT1	Biolegend
**CD21-BV605**	1048	BD bioscience
**CD23-PE**	EBVCS-5	Biolegend
**CD27-PE-Dazzle594**	O323	Biolegend
**CD24-PercpCy5.5**	ML5	Biolegend
**CD19-PE-Cy7**	HIB19	Biolegend
**CD38-Alexa-Fluor-700**	HB-7	Biolegend
**CD20-APC-Cy7**	2H7	Biolegend
**Ara-H-2 Pacific Blue**		
**Ara-H-2 Alexa-Fluor-488**		
	**Intracellular Staining**	
**Ki67 APC**	REA183	Miltenyi

T cell panel 1

	**Surface Staining**	
**CCR4-BV510**	L291H4	Biolegend
**CD8-BV650**	RPA-T8	Biolegend
**CXCR5-PE-Vio615**	REA103	Miltenyi
**CD4-PERCP**	RPA-T4	Biolegend
	**Intracellular Staining**	
**IL-5-BV521**	TRFK5	Biolegend
**CD154-BV605**	24-31	Biolegend
**FOXP3-Alexa-Fluor-488**	259D	Biolegend
**CD137-PE**	REA765	Miltenyi
**IL-4-PE-Vio-770**	REA895	Miltenyi
**Helios-Alexa-Fluor-647**	22F6	Biolegend
**IFNy-Alexa-Fluor-700**	4S.B3	Biolegend
**IL-17A-APC-Vio-770**	REA1063	Miltenyi

T cell panel 2

	**Surface Staining**	
**PD1-BV421**	EH12.2H7	Biolegend
**CCR4-BV510**	L291H4	Biolegend
**CD8-BV650**	RPA-T8	Biolegend
**CTLA4-PE-Dazzle-594**	BN13	Biolegend
**CD4-PERCP**	RPA-T4	Biolegend
**CD38-APC**	REA572	Miltenyi
**HLADR-APC-Vio770**	REA805	Miltenyi
	**Intracellular Staining**	
**KI67-Alexa-Fluor-488**	Ki-67	Biolegend
**CD154-BV605**	24-31	Biolegend
**CD137-PE**	REA765	Miltenyi
**IL-4-PE-Vio-770**	REA895	Miltenyi
**IFNy-Alexa-Fluor-700**	4S.B3	Biolegend

Supplementary Figure S1

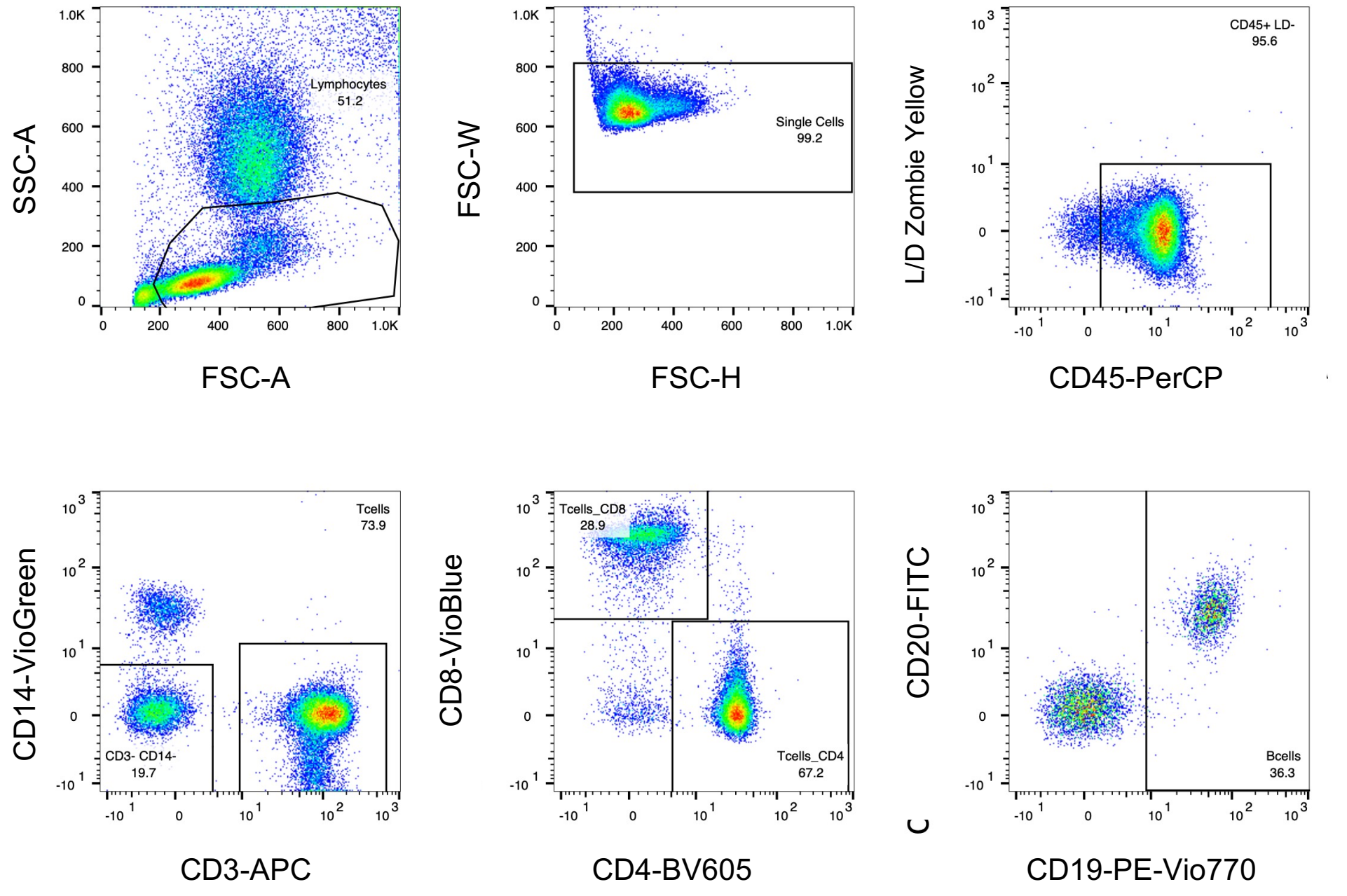

Supplementary Figure S1: Gating scheme for whole blood count panel.
Whole blood samples were stained with the Whole blood count panel (Supplementary Table S1). Events were gated for FSC-W vs. FSC-H to exclude doublets, and LD ZombieYellow to discriminate dead cells. Alive cells were gated for CD45 to identify lymphocytes, which were further separated into CD3+ T cells, then CD4+ and CD8+ T cells. CD3- lymphocytes were gated for CD19+ CD20+ B cells.

Supplementary Figure S2

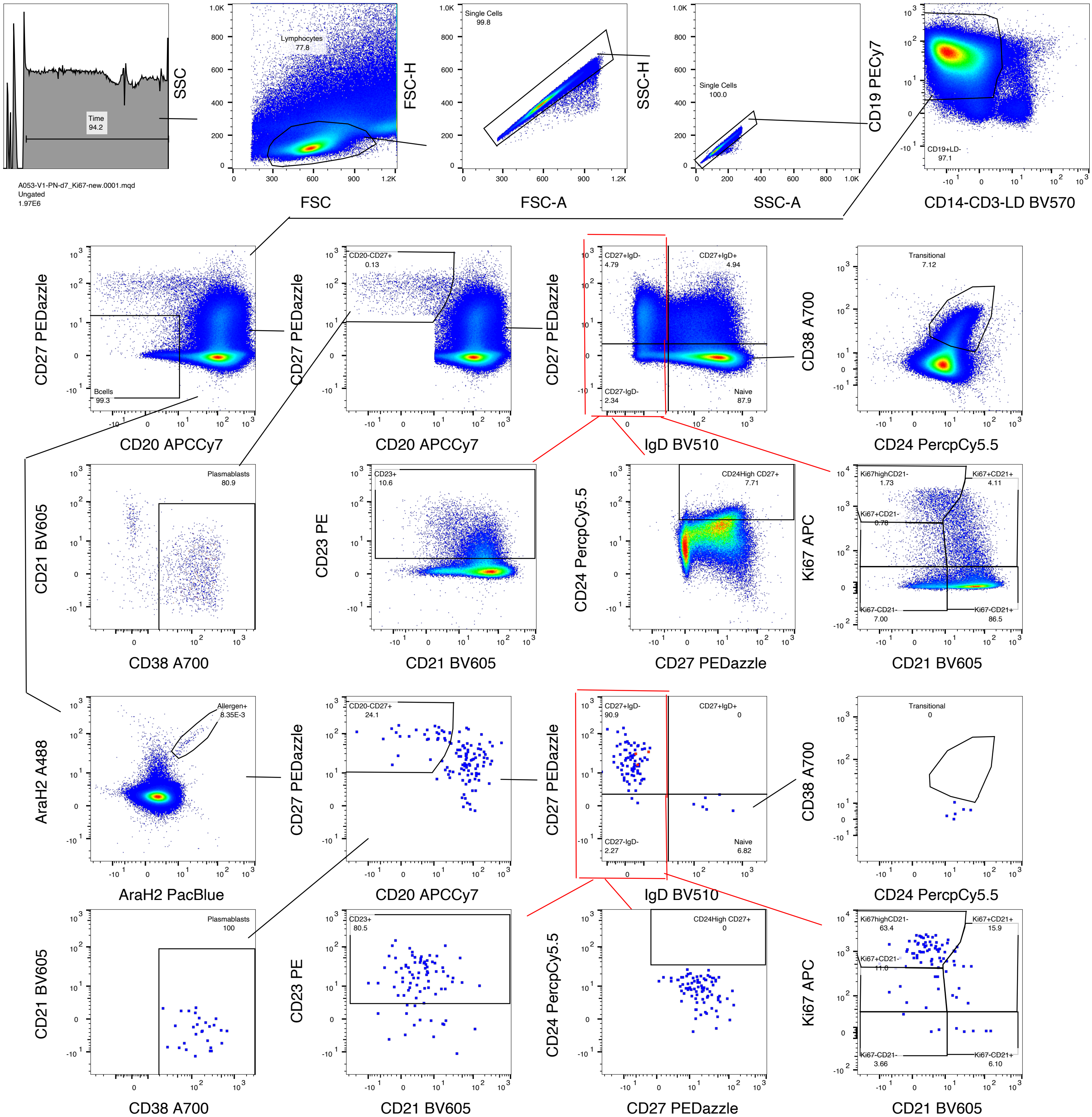

Supplementary Figure S2: Gating scheme for Ara h2-specific B cells and memory subsets. CD19-enriched PBMCs were stained with the B cell panel (Supplementary Table 1). Events were gated for FSC-W vs. FSC-H and SSC-W vs. SSC-H to exclude doublets, and for CD19 vs. CD14, CD3 and LD ZombieYellow to gate for B cells and exclude dead cells, T cells and monocytes. Alive, bulk B cells were identified as CD20^{+} and CD27^{+} and investgated further (2nd and 3rd row). Among CD20^{-} CD27^{+} cells, CD21^{-} CD38^{+} cells were gated as plasmablasts. Among CD20^{+} B cells, memory subsets were gated using CD27 and IgD. IgD^{-} memory B cells (red box) were further characterized using CD23, CD24 and Ki-67 vs. CD21. Among naïve IgD^{+} CD27^{-} B cells, transitional B cells were gated as CD38^{+} CD24^{+} . Allergen-specific B cells were identified among bulk B cells by dual color labeled Ara h 2 and analyzed anlogous to bulk B cells (4th and 5th row).

Supplementary Figure S3

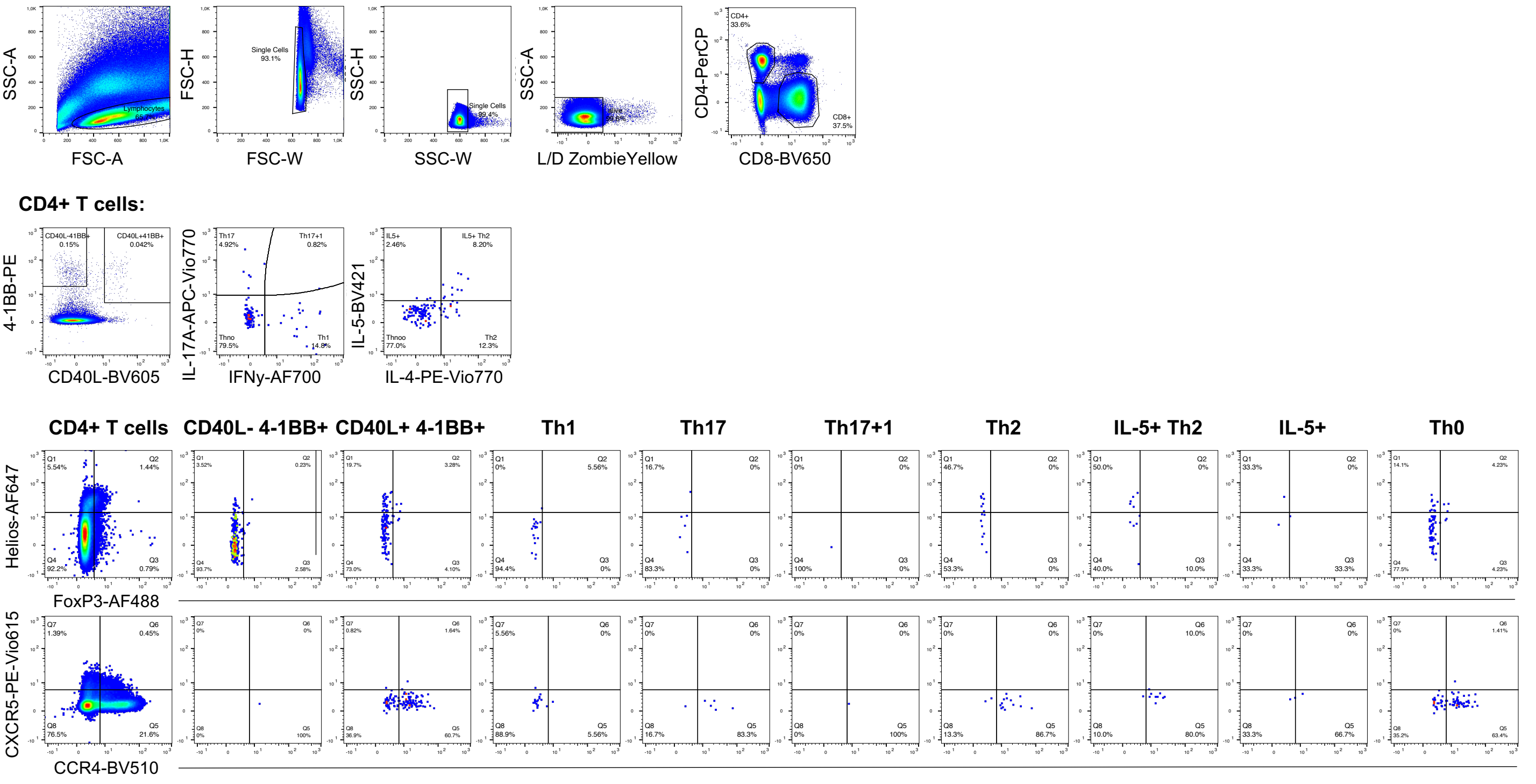

Supplementary Figure S3: Gating scheme for antigen-specific T cells, helper subsets and homing markers.

After stimulation, CD19- PBMCs were stained with the T cell panel 1 (Supplementary Table 1). Events were gated for FSC-W vs. FSC–H and SSC-W vs. SSC–H and LD ZombieYellow to exclude doublets and dead cells. Alive PBMCs were separated into CD8+ and CD4+ T cells, and in the latter, activated cells were assessed as CD40L^{-}4-1BB^{+} and CD40L^{+} 4-1BB^{+} to tdentify regulatory and conventional effector T cells. The latter were further separated based on IFNg, IL-4, IL-5 and IL-17A staining into Th1 (IFNg^{+} IL-17A^{-}), Th2 (IL-4^{+} IL-5^{-}), IL-5^{+} Th2 (IL-4^{+} IL-5^{+}), IL-5^{+} (IL-5^{+} IL-4^{-}), Th17 (IL-17A^{+} IFNg^{-}), Th17+1 (IL-17A^{+} IFNg^{+}) and Th0 (IFNg^{-} IL-4^{-} IL-5^{-} IL-17A^{-}). Within these populations, cells were characterized for FoxP3 and Helios (upper row) as well as CXCR5 and CCR4 expression (lower row). Within CD40L^{-} 4-1BB^{+}, FoxP3^{+} Helios^{+} were gated as regulatory T cells (Tregs) and CXCR5 and CCR4 are analysed within this subpopulation. Example of a PN-stimulated sample is depicted.

Supplementary Figure S4

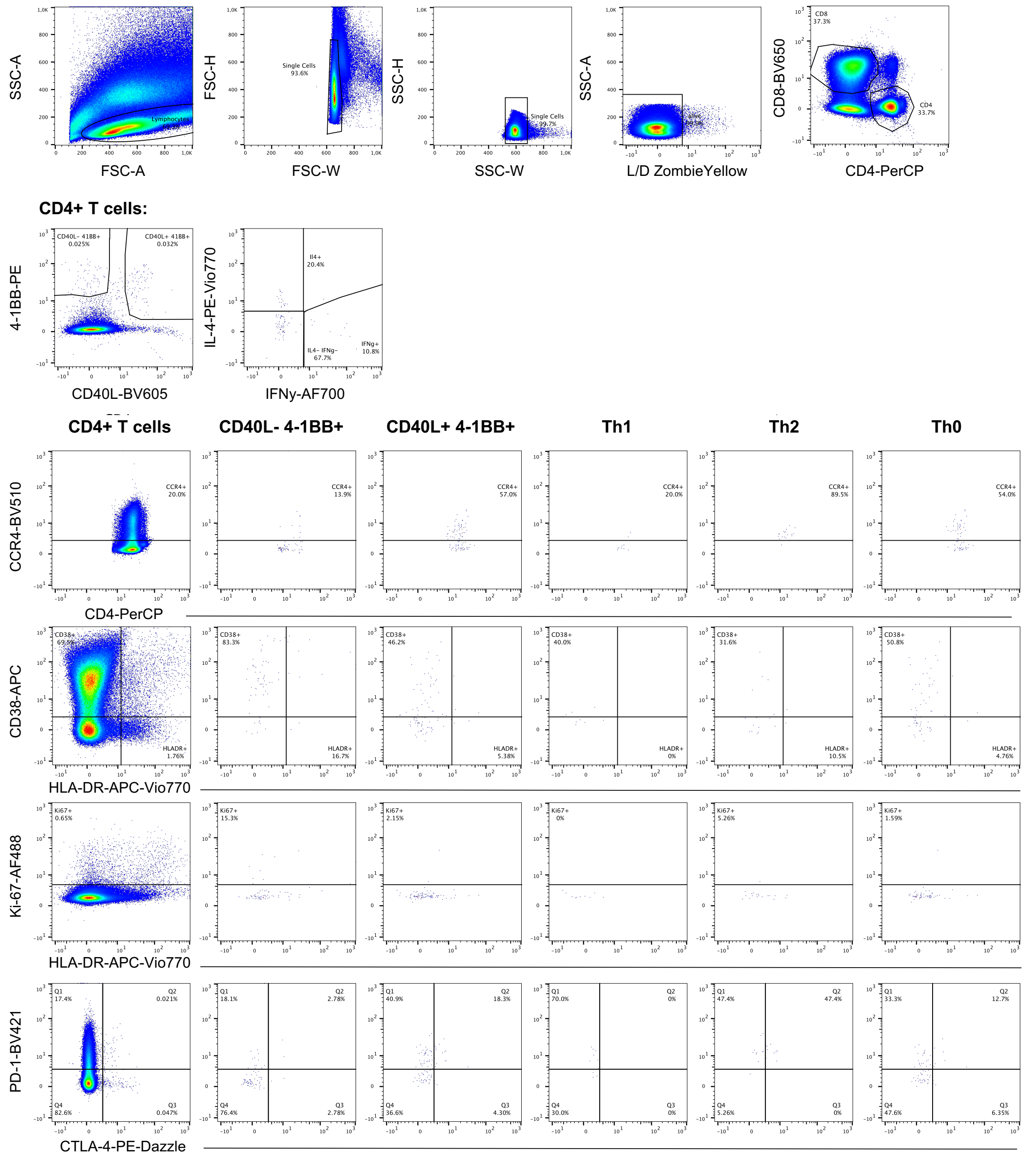

Supplementary Figure S4: Gating scheme for T cell activation, in vivo activation markers and checkpoint inhibitors.

After stimulation, CD19- PBMCs were stained with the T cell panel 2 (Supplementary Table 1). Evemts were gated for FSC-W vs. FSC-H and SSC-W vs. SSC-H and LD ZombieYellow to exclude doublets and dead cells. Alive PBMCs were separated into CD8+ and CD4+ T cells, and in the latter activated cells were assessed as CD40L$^-$ 4-1BB$^+$ and CD40L$^+$ 4-1BB$^+$ to tdentify regulatory and conventional effector T cells. The latter were further separated into Th1 (IFNg$^+$) and Th2 (IL-4$^+$). Within these populations, cells were characterized for CCR4, HLA-DR, CD38, Ki-67, PD-1 and CTLA-4 expression. Example of PN-stimulated sample is depicted.

Supplementary Figure S5

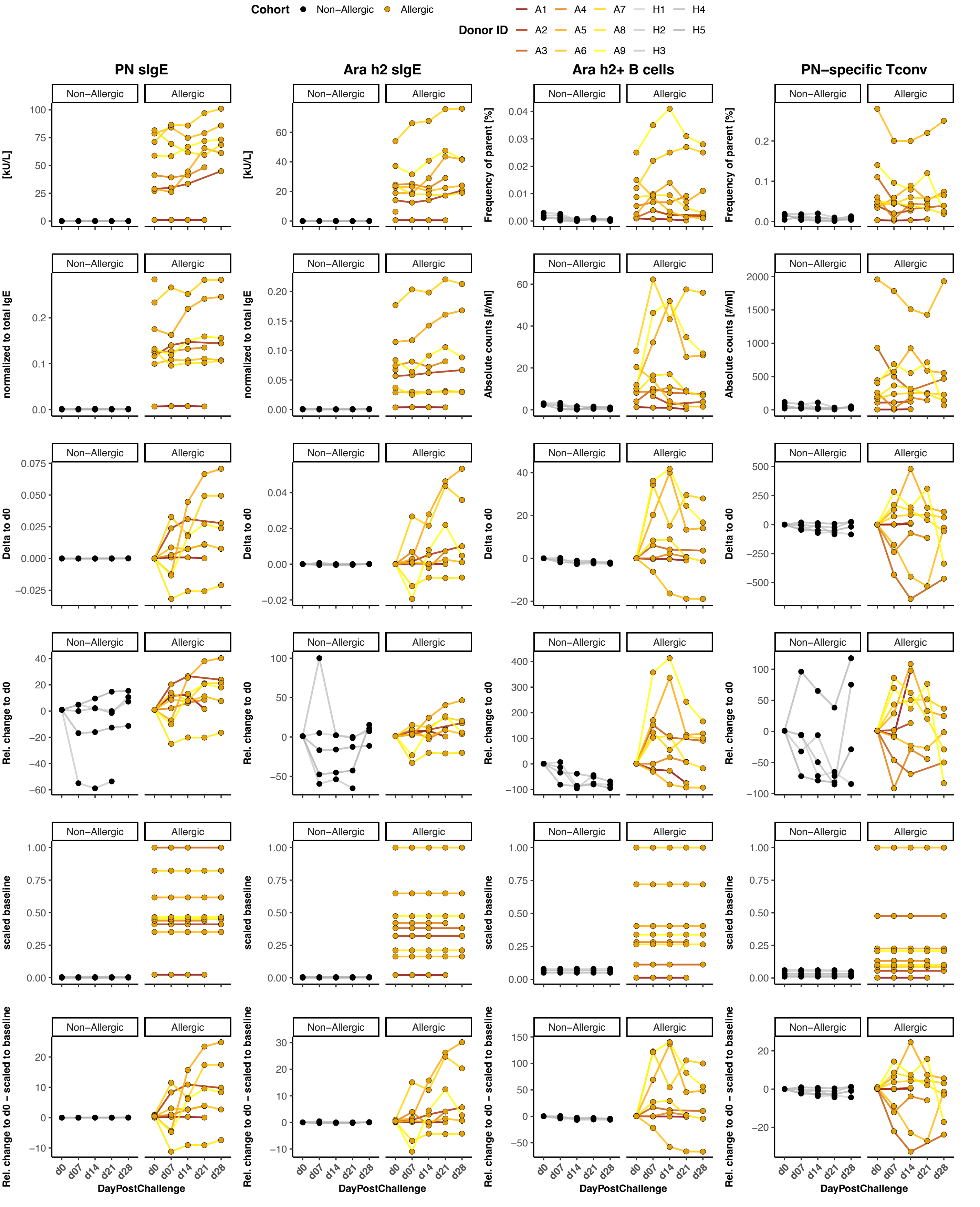

Supplementary Figure S5: Derivatives to capture dynamic regulations.
Comparison of different calculated derivatives are depicted for exemplary parameters, namely PN sIgE, Ara h 2 sIgE, Ara h 2+ B cells and PN-specific Tconv as kU/L or frequency of parent (1st row) or normalized to total IgE or absolute counts (2nd row). Three derivatives were calculated: 1) "Delta to d0", the difference of any timepoint to d0 (3rd row); 2) Rel. change, the change relative to d0, i.e. the delta divided by the baseline value (4th row); 3) Baseline normalization factor , generated across baseline values (5th row); and 4) Scaled Rel. change, the Rel. change was normalized per parameter by multiplication with the baseline normalization factor (6th row).

Supplementary Figure S6

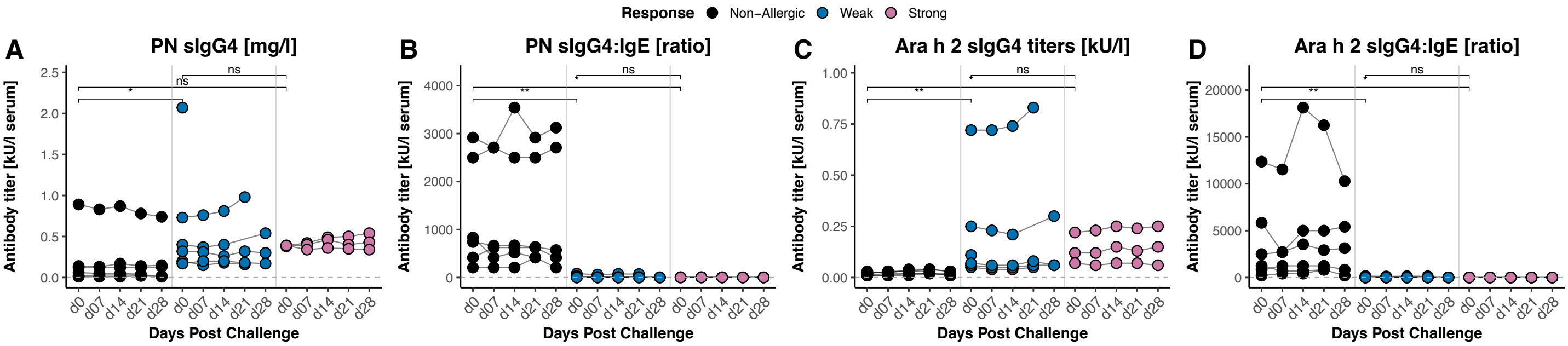

Supplementary Figure S6: Overview of IgG4 serology.
PN as well as Ara h 2-specific IgG4 and ratio of IgG4 / IgE are displayed, separated for non-allergic and the different response patterns among allergic donors. Statistical comparisons were performed using Wilcoxon test (°, $p < 0.1$; *, $p<0.05$; **, $p<0.01$; ***, $p<0.001$; ns, not significant).

Supplementary Figure S7

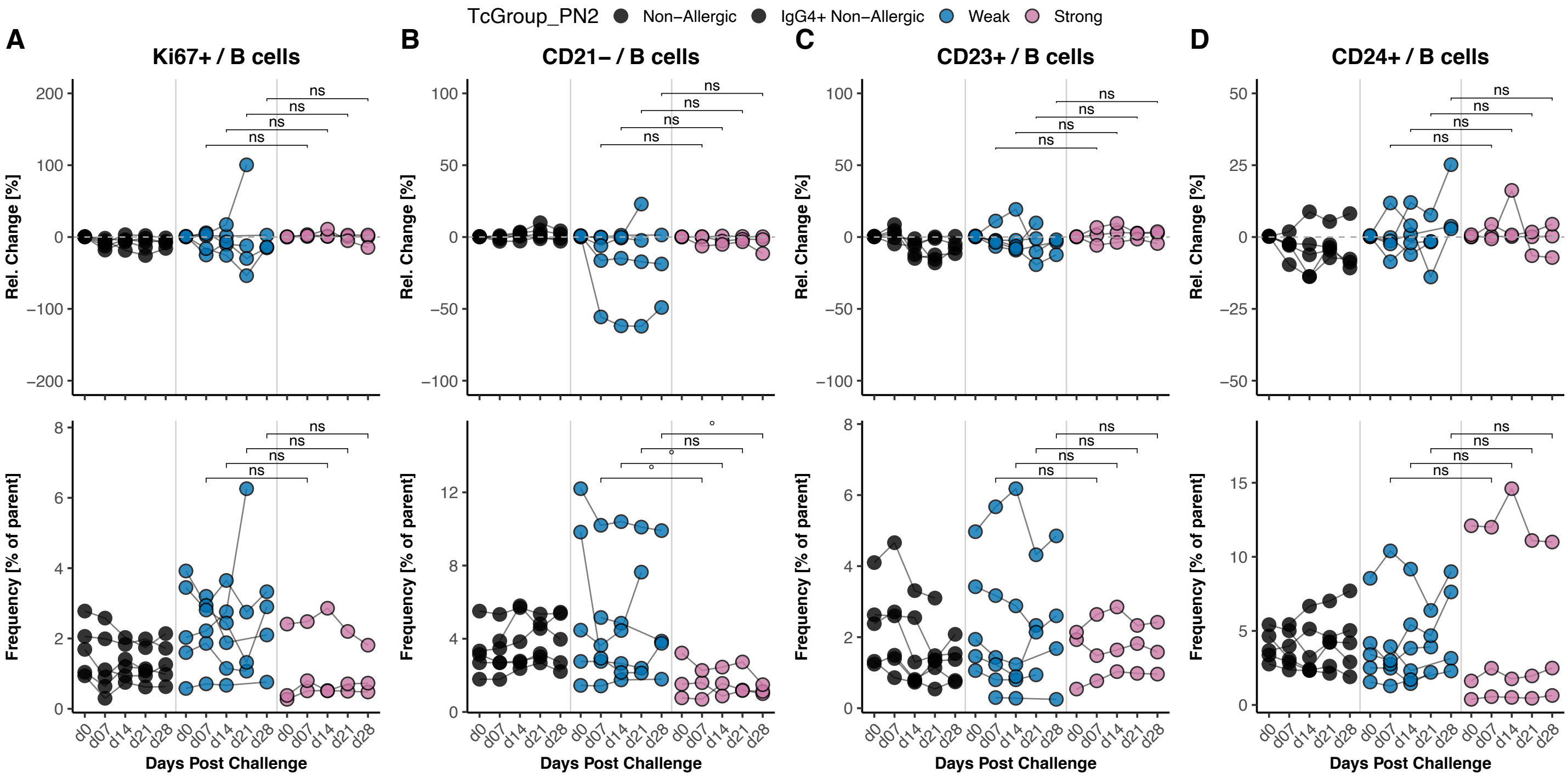

Supplementary Figure S7: Dynamics of selected bulk B cell populations.

A-D: Scaled relative change (upper row) and absolute counts or frequency of parent (lower plot) of indicated cell populations over time displayed for non-allergic donors (black) and allergic Weak (blue) and Strong (pink) responders. Statistical comparisons were performed using Wilcoxon test (°, $p < 0.1$; *, $p<0.05$; **, $p<0.01$; ***, $p<0.001$; ns, not significant).

Supplementary Figure S8

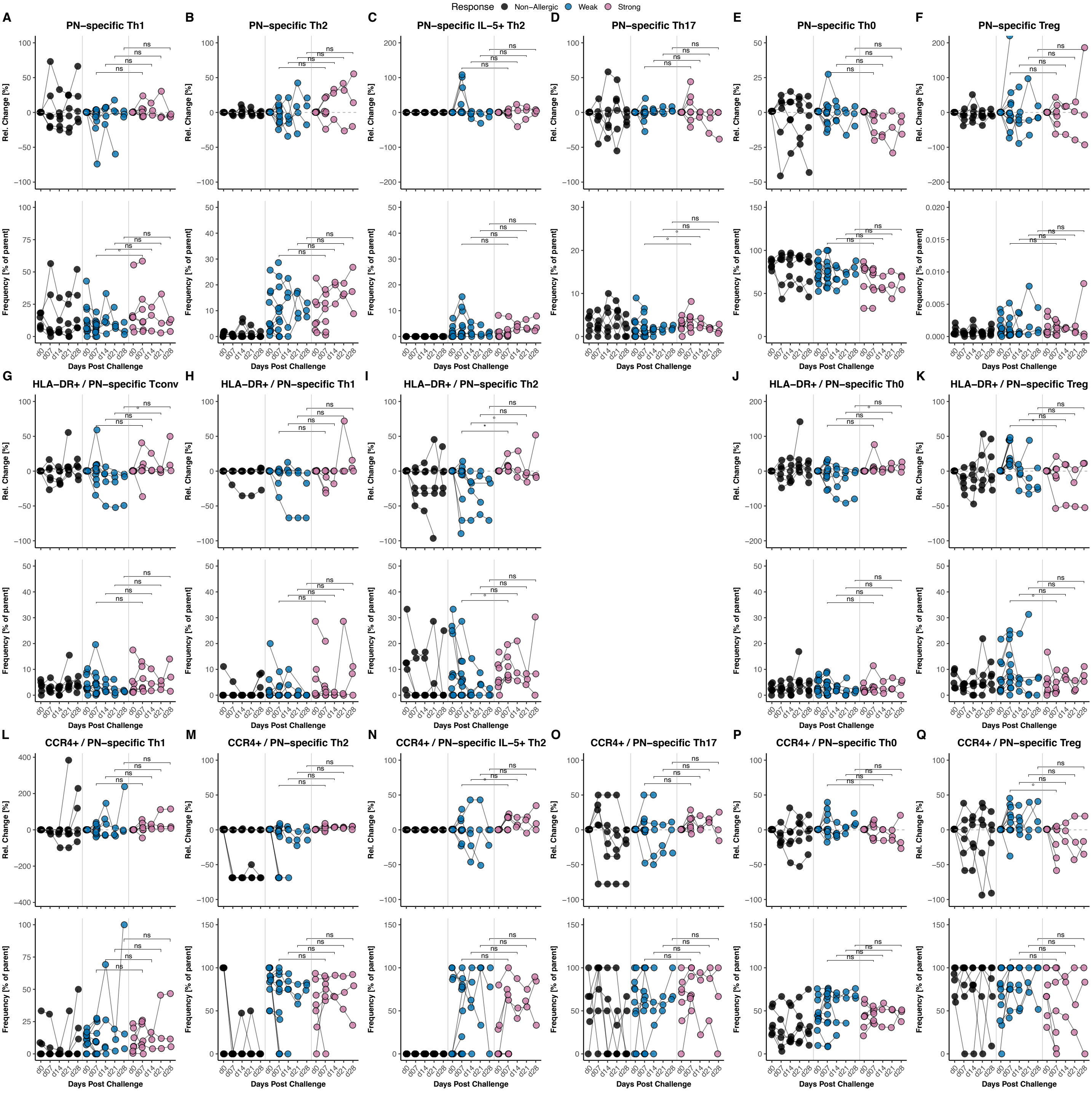

Supplementary Figure S8: Dynamics of relevant T cell subsets.

A-O: Scaled rel. change (upper plot) and absolute counts or frequency of parent (lower plot) of indicated cell populations over time displayed for non-allergic donors (black) and allergic Weak (blue) and Strong (pink) responders. Statistical comparisons performed using Wilcoxon test (°, $p < 0.1$; *, $p<0.05$; **, $p<0.01$; ***, $p<0.001$; ns, not significant).

Supplementary Figure S9

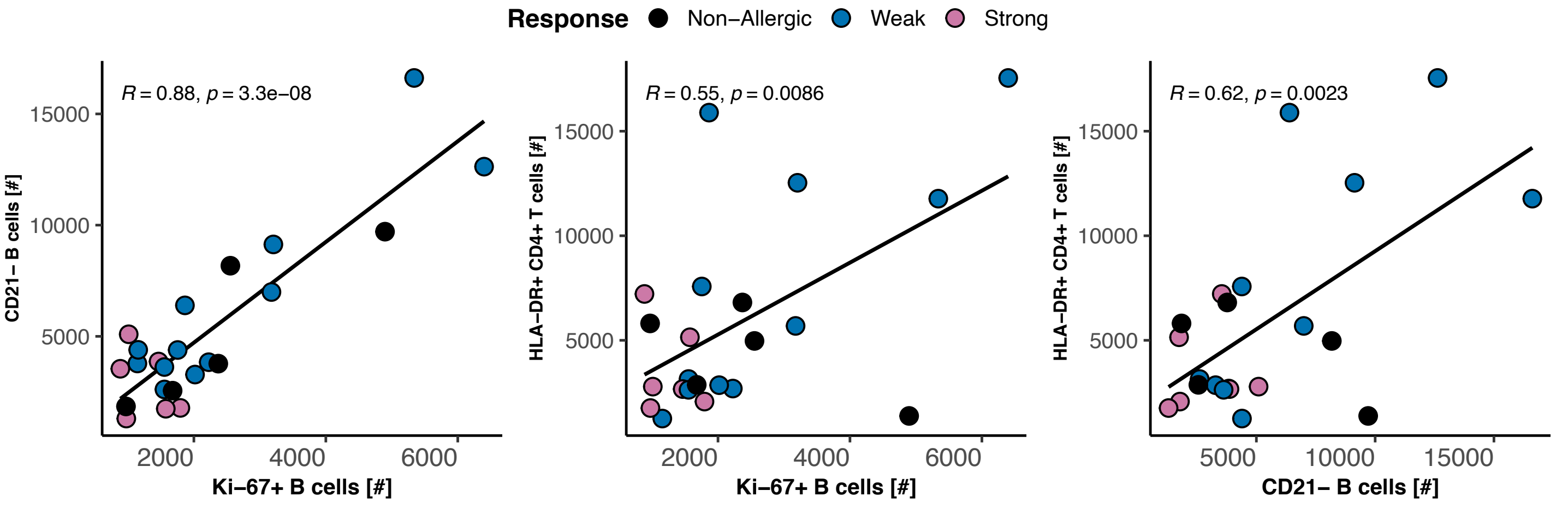

Supplementary Figure S9: Correlation of activated B cells and T cells points at general immune activation.

Spearman correlations are shown for absolute counts of Ki-67+ B cells, CD21- B cells and HLA-DR+ CD4+ T cells. Dots are tinted for non-allergic donors (black), and allergic Weak (blue) and Strong (pink) responders. Correlation coefficient R and significance level p of Spearman correlations are shown in graph. Regression line in black.

Supplementary Figure S10

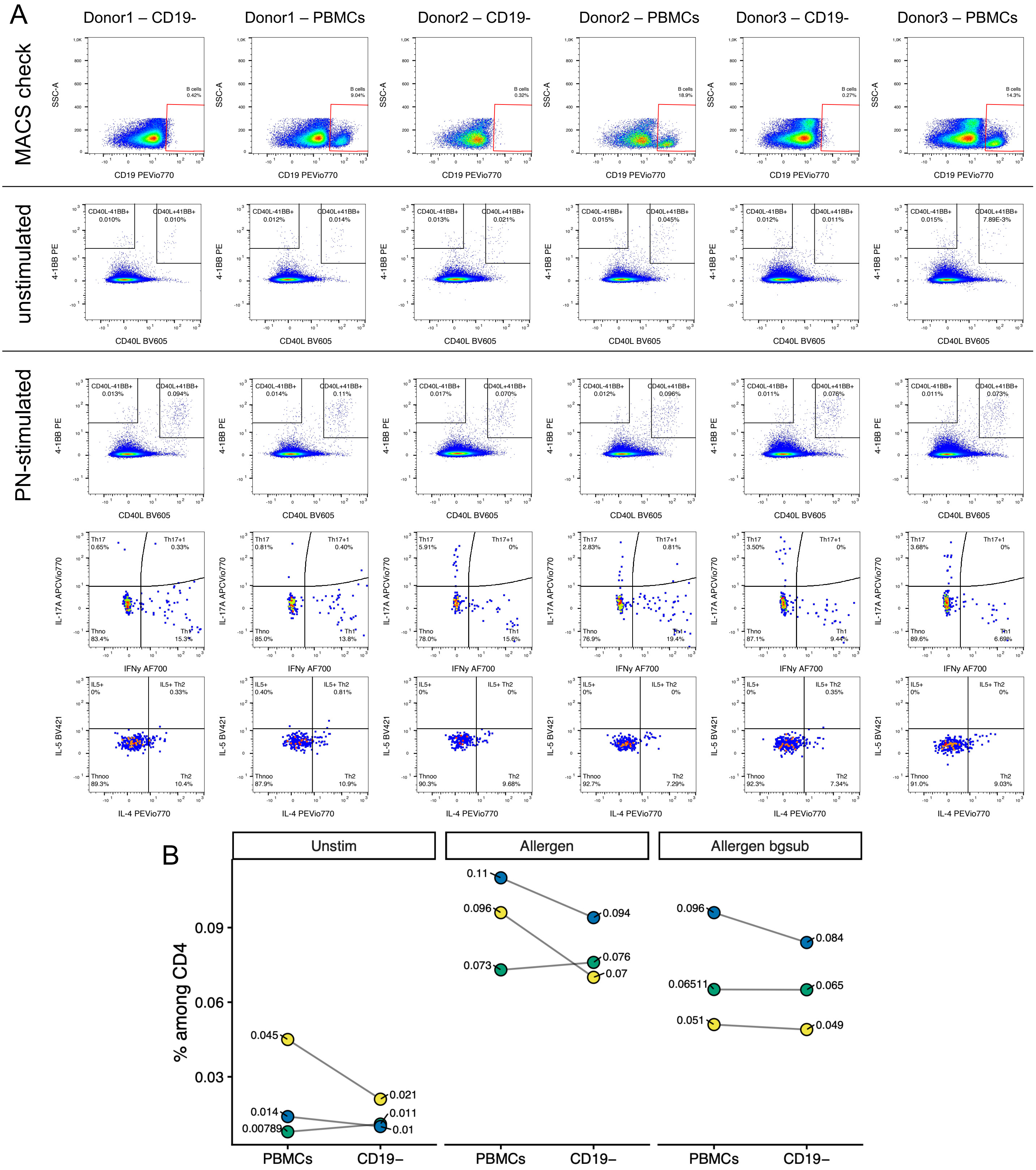

Supplementary Figure S10: Impact of B cell removal on T cell stimulation assay.
Three PN-allergic donor samples (PBMCs) were B cell depleted using anti-CD19 magnetic beads ($CD19^-$) and stimulated with PN extract to determine PN-reactive T cells in the presence or absence of B cells. A: Dot plots showing SSC-A vs. CD19 illustrate the high purity of the B cell depletion (MACScheck). Frequencies of $CD40L^+4\text{-}1BB^+$ and $CD40L^-4\text{-}1BB^+$ $CD4^+$ T cells in the unstimulated control as well as in the PN-stimulated sample differ to some extent between the PBMCs and the $CD19^-$ PBMCs. Frequencies of cytokine-producing T cells among $CD40L^+4\text{-}1BB^+$ $CD4^+$ T cells are not different between PBMCs and $CD19^-$ (lower two rows). B: Graphic representation of frequencies of $CD40L^+$ $4\text{-}1BB^+$ among $CD4^+$ T cells in the unstimulated and PN-stimulated samples and after background subtraction.
